# Caution on the severe damage of *Gelsemium elegans* poisoning: a case report on family poisoning and systematic review

**DOI:** 10.3389/fpubh.2025.1633727

**Published:** 2025-08-11

**Authors:** Yawen Zhao, Bisheng Shen, Qimei Xiao, Yameng Wang, Yi Fu, Qi Tang, Zhangrong Liang

**Affiliations:** 1 The Eighth Clinical Medical College of Guangzhou University of Chinese Medicine, Foshan, China; 2 Foshan Hospital of Traditional Chinese Medicine, Foshan, China

**Keywords:** *Gelsemium elegans*, poisoning, case report, review-systematic, gastric lavage

## Abstract

**Objective:**

This study reports a familial Gelsemium elegans poisoning case and systematically evaluates the efficacy of gastric lavage in *G. elegans* intoxication through a meta-analysis.

**Methods:**

We systematically searched PubMed, Web of Science, CNKI, and Wanfang Data (2000–2024). Eligible randomized controlled trials and case reports comparing gastric lavage versus non-lavage approaches were included. Data were extracted from published studies, and pooled odds ratios (ORs) with 95% confidence intervals (CIs) were calculated using Mantel–Haenszel random-effects models.

**Results:**

Thirteen studies involving 160 patients demonstrated an overall in-hospital mortality of 18.75%. Gastric lavage suggested potential survival benefit compared to controls (OR 0.39; 95% CI 0.15–0.99; *p* = 0.62).

**Conclusion:**

*G. elegans* poisoning is life-threatening, with severe cases rapidly progressing to respiratory/circulatory failure requiring urgent support. Gastric lavage may offer survival advantage in hemodynamically stable patients when performed with airway protection. Prompt respiratory support should be prioritized in the therapeutic management.

## Introduction

**Figure 1:**
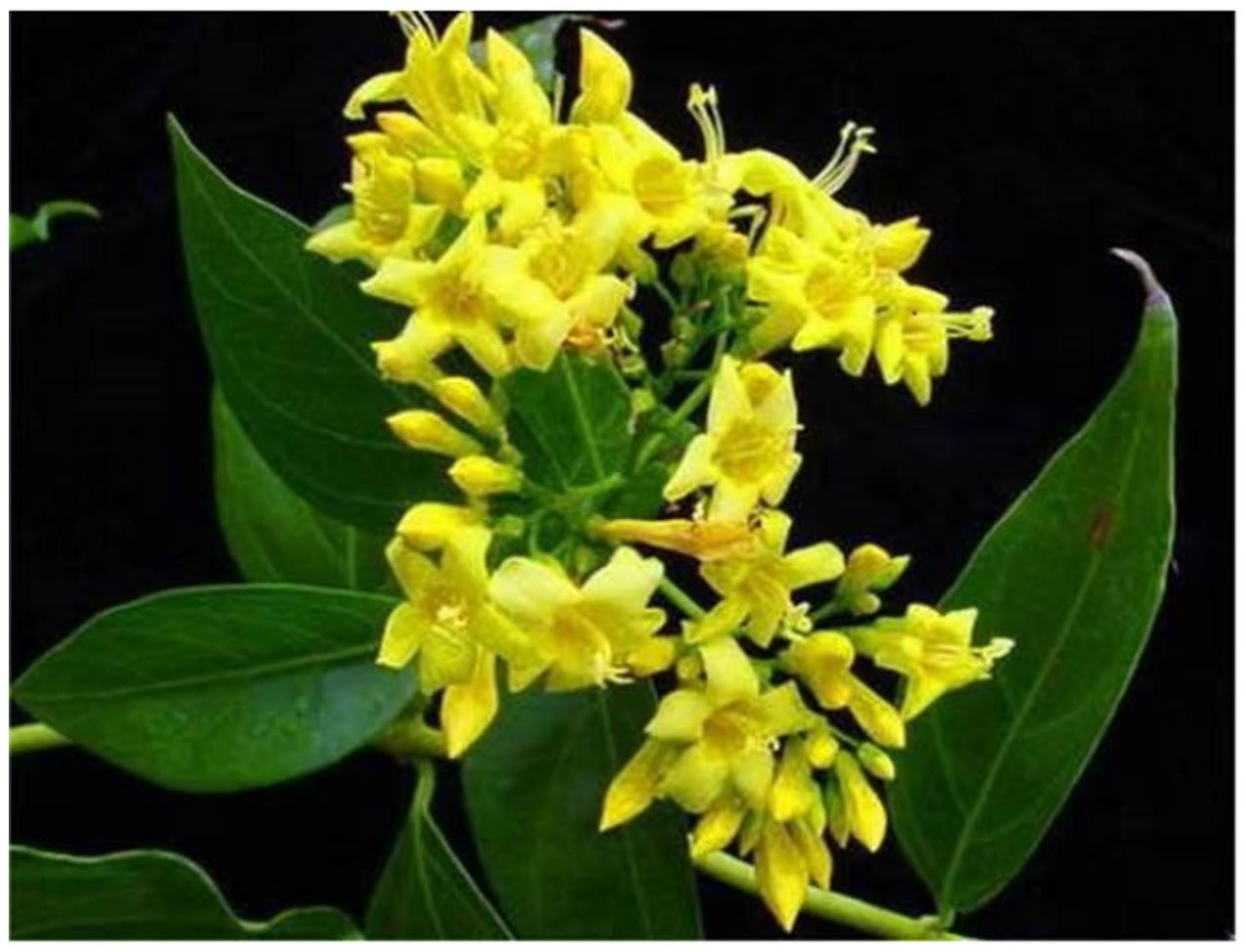
Flowers and leaves of *Gelsemium elegans.*

**Figure 2:**
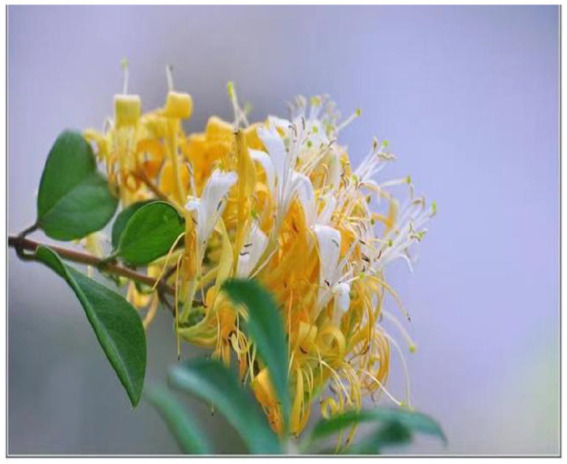
Flowers and leaves of *Lonicera japonica*. Pictures courtesy of Mr. Li Xibeiyang and Plant Photo Bank of China. *Lonicera japonica* is a semi-evergreen climbing shrub characterized by tubular flowers (3–4.5 cm in length) that transition from white to yellow coloration. The flowering period primarily occurs from April to June (with frequent autumn flowering periods observed), featuring ovate to oblong-ovate leaf morphology. *Lonicera japonica* predominantly inhabits montane shrublands at elevations below 1,500 meters, demonstrating notable adaptability to various soil conditions. This plant serves as a fundamental herb in traditional Chinese medicine, where the medicinal components are derived from either dried flower buds or partially opened flowers at early developmental stages.

**Figure 3:**
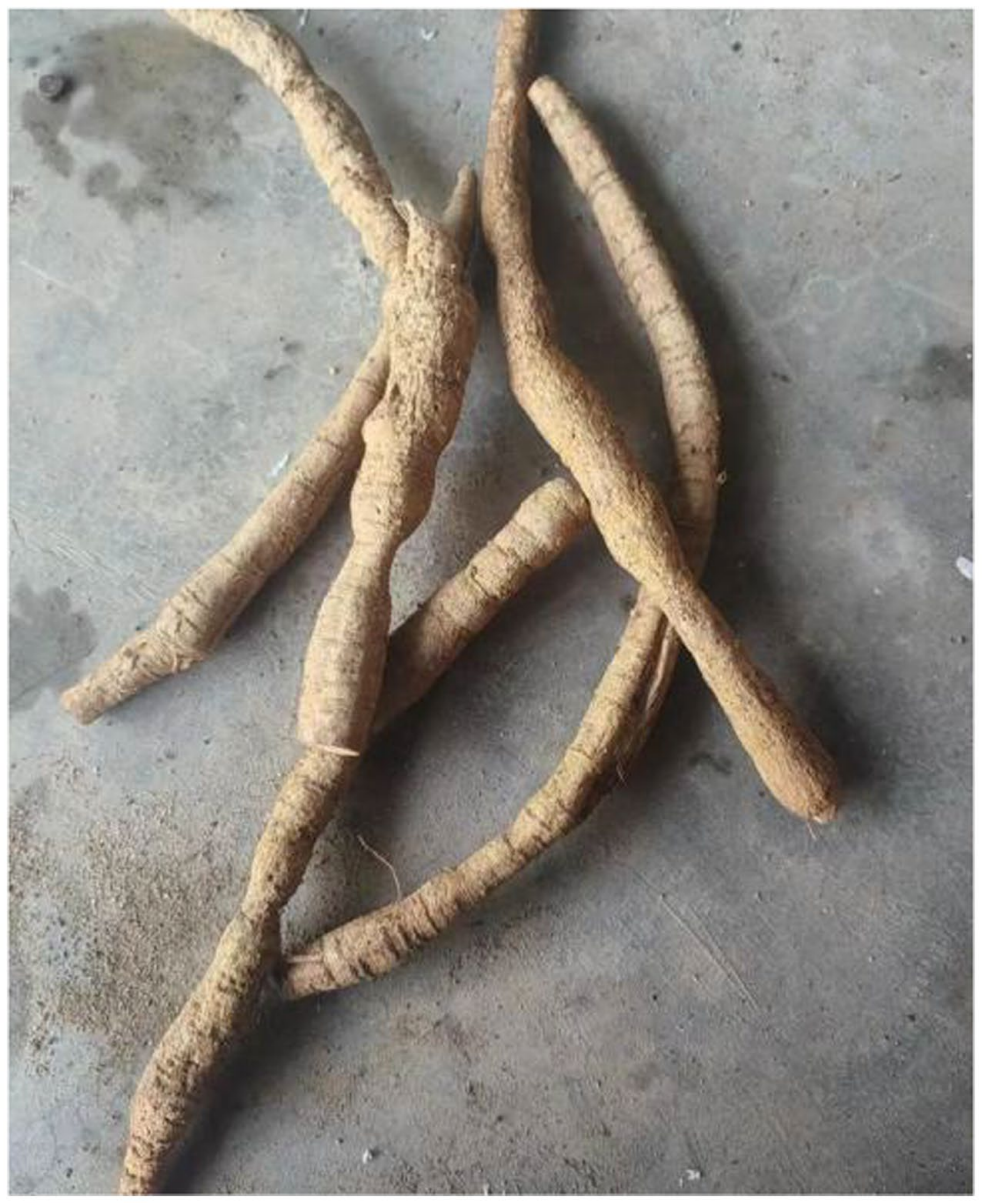
Roots from radix Millettiae Speciosae. Millettia speciosa (commonly called Cattle Strength Vine) is a climbing shrub with thick, fleshy roots that look like twisted intestines or strings of irregular beads. These roots are juicy but contain tough fibers. Primarily found in southern China, it grows in provinces including Guangdong, Guangxi, Yunnan, Fujian, Hunan, Guizhou, and Hainan. This plant thrives in various environments such as valleys, roadsides, shrublands, and open forests. The dried roots are used in traditional medicine and can be collected any time of year. Source from https://www.douyin.com/note/7384251535108148492

*Gelsemium elegans* (*G. elegans*) is a highly toxic plant that contains various poisonous alkaloids (Figure 1) (1). It was found in a broad range of geographic regions, with reports of its presence in Southeast Asian countries and Chinese southern provinces (2). History reports that Shen Nong was fatally poisoned b *G. elegans*, which led to the plant being referred to as “Duan Changcao”in Chinese (1). Due to its resemblance to several traditional Chinese medicines with health benefits, such as *Lonicera japonica* (Figure 2) and *Radix Millettiae Speciosae* (Figure 3), accidental ingestion can occur, leading to acute poisoning caused by mistaken ingestion (3). This report describes three successfully treated cases of *G. elegans* poisoning. And through a systematic review we evaluated the clinical impact of gastric lavage on outcomes in *G. elegans* poisoning while synthesizing reported clinical presentations, treatment modalities, and outcome determinants.

## Case history

**Figure 4:**
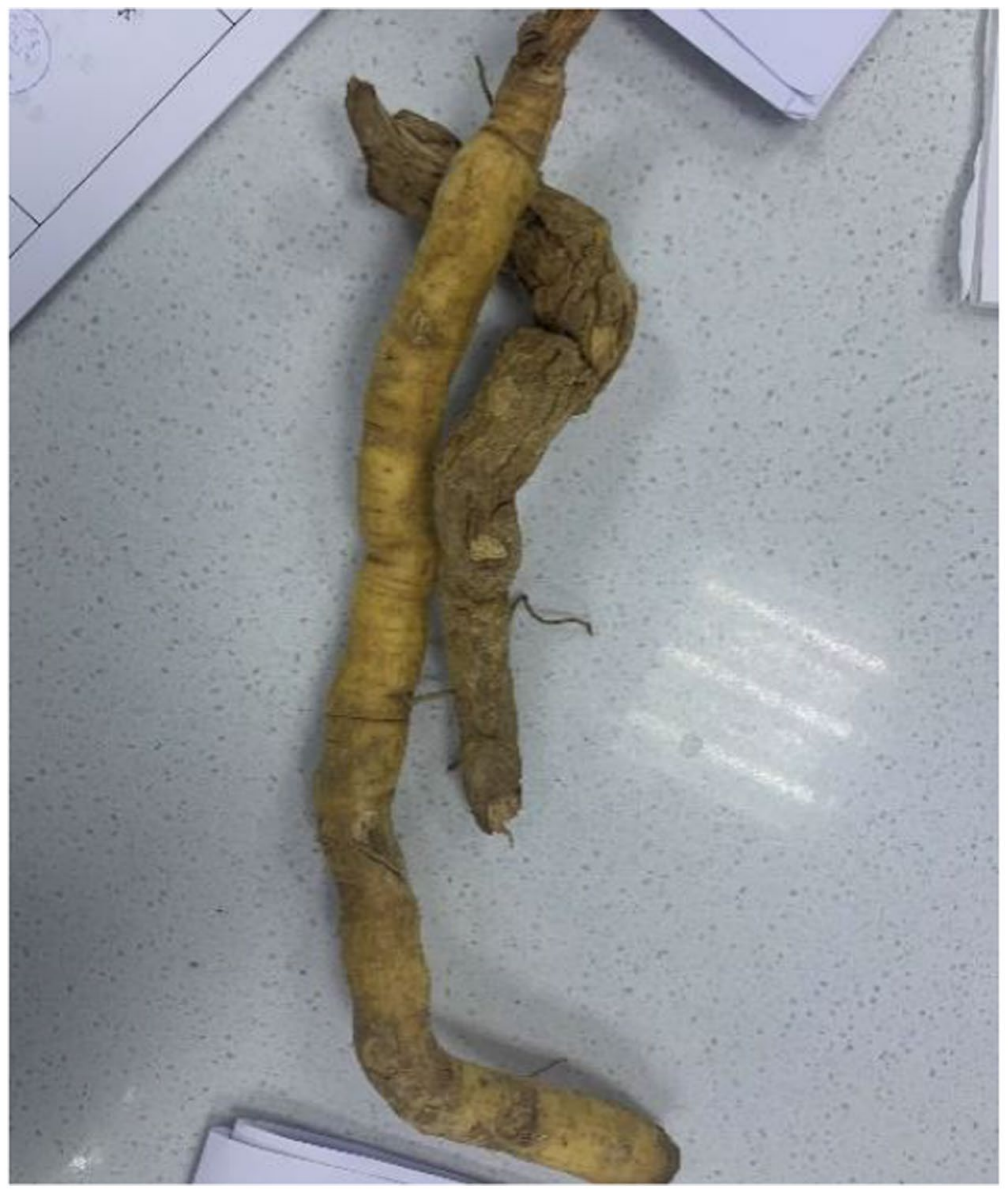
Roots of *Gelsemium elegans* from scene investigation.

Three family members ingested a home-prepared herbal broth at approximately 7:00 PM. All individuals manifested symptoms of dizziness, visual disturbance, generalized numbness with weakness, and dyspnea following ingestion of herbal soup. Emergency medical services (EMS) were contacted at 19:20, with personnel arriving promptly to collect samples (Figure 4) within 30 min. All patients were transferred back to emergency department of Foshan Hospital of Traditional Chinese Medicine for continued resuscitation and therapeutic management.

### Case 1

A 55-year-old male, who was healthy all along. He presented with symptoms of dizziness, weakness, blurred vision, and shortness of breath about 20 min after ingestion. Upon admission, his Glasgow Coma scale was 13/15, blood pressure 144/96 mmHg, respiratory rate of 30 breaths/min, pulse rate of 100 bpm, and blood oxygen saturation level of 100%. His dyspnea worsened progressively, accompanied by unconsciousness and cyanosis of the lips and fingernails at 20:10. Due to acute respiratory failure, the patient was intubated and transferred to the emergency intensive care unit (EICU). Arterial blood gas analysis revealed pH 7.395, PaO2 182.3 mmHg, PaCO_2:_36.5 mmHg, CO2 22.3 mmol/L, BE:-2.5 mmol/L, K^+^3.7 mmol/L, Glu:7.8 mmol/L, SO2 55%, lactate 4.2 mmol/L.

### Case 2

A 49-year-old previous heathy female, was the wife of case 1. She developed symptoms of blurred vision, peripheral numbness with weakness, and nausea by accompanied emetic tendency about 20 min post-ingestion. Her Glasgow Coma scale was 14/15, blood pressure 133/76 mmHg, respiratory rate of 20 breaths/min, pulse rate of 98 bpm, and temperature 36.8°C. Both her pupils were 3 mm in size and were reactive to light. The limb muscle power was 3/5 and deep tendon reflexes were normal. At 20:42, the patient developed somnolence with intermittent limb convulsions accompanied by a decline in oxygen saturation (SpO_2_) to 68%. Endotracheal intubation with mechanical ventilation was subsequently initiated, followed by ICU admission for continuous intensive monitoring and therapeutic management.

### Case 3

A 33-year-old woman with history of Sjögren’s syndrome, was the daughter in law of case 1. Her clinical manifestations resembled those observed in Case 2, with concomitant development of generalized erythema. Her Glasgow Coma scale was 13/15, blood pressure 119/86 mmHg, respiratory rate of 24 breaths/min, pulse rate of 115 bpm, and temperature 36.5°C. Both her pupils were 3 mm in size and were reactive to light. At 20:40, the patient developed a comatose state accompanied by intermittent limb convulsions and frothy oral secretions. Endotracheal intubation was emergently performed with subsequent initiation of mechanical ventilation. The patient was transferred to the intensive care unit (ICU) for advanced life support and ongoing critical care management.

Given the short poisoning interval (30 min), the medical team performed emergent gastric lavage (4) and hemoperfusion was subsequently initiated to enhance toxin elimination following admission to the EICU (5). Laboratory findings revealed leukocytosis in all patients (Case 1: 11.15 × 10^9^/L; Case 2: 11.73 × 10^9^/L; Case 3: 13.76 × 10^9^/L), accompanied by elevated serum amyloid A (SAA) levels. There were no notable abnormalities in the patients’ coagulation function, cardiac enzymes, chest X-ray, and brain CT scan. Adjunctive therapies including anti-infection, gastrointestinal protection and correction of electrolyte imbalances. All patients regained spontaneous respiration by hospital day 2 and were discharged after a five-day treatment period without any neurological complications.

**Figure 5:**
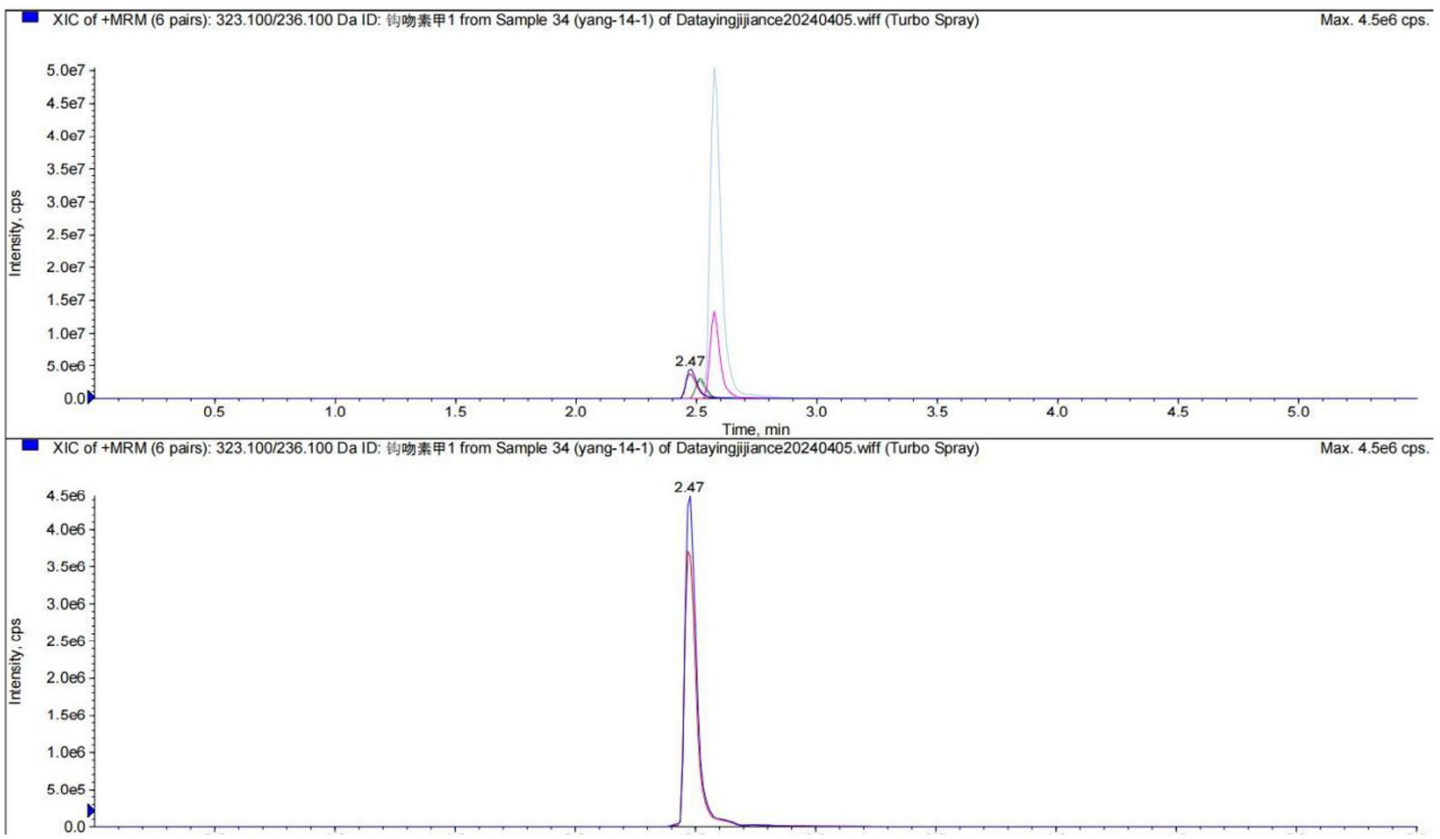
The report of liquid chromatography from the FoShan Center for Disease Control and Prevention (FoShan CDC).

On April 7, the Foshan Center for Disease Control and Prevention (CDC) confirmed the cause of poisoning as *G. elegans* through liquid chromatography analysis of food samples collected on-site (Figure 5).

## Materials and methods

### Database search strategy

Literature screening and data analysis were conducted in accordance with PRISMA guidelines (6). The study protocol has been resisted in the PROSPERO database (Registration number: 2025 CRD420251027380). The scope of the review was to answer the following PICO question: “In patients with acute *Gelsemium elegans* poisoning, does gastric lavage compared with supportive care without gastric lavage reduce mortality and incidence of long-term sequelae?.” The primary research question was formulated according to the PICO framework (P: population, I: intervention, C: comparator, O: outcome; Table 1). A comprehensive literature search, encompassing databases Web of Science, PubMed, China National Knowledge Infrastructure (CNKI), and Wanfang Data has been performed with the following Boolean Indicators: (“*Gelsemium*” OR “*gelsemium* poisoning” Table 2).

**Table 1:** Summary of the PICO question considered for the systematic review.

Procedure			
Population/Patients	Intervention	Comparison	Outcomes
Subjects with acute *Gelsemium elegans* poisoning	Gastrointestinal decontamination	No gastrointestinal decontamination	Mortality Incidence of long-term sequelae

**Table 2:** Summary of the search strategy applied for the electronic database search.

Databases	Search Strategies
Web of Science, PubMed, China National Knowledge Infrastructure (CNKI), and Wanfang Data	*“Gelsemium*” OR “gelsemium poisoning”

### Inclusion/exclusion criteria

The inclusion criteria specified clinical trials, case reports or case series on patients with no follow up limitations. The exclusion criteria were limited to animal studies, systematic reviews, short communications and subjects who died prior to hospital admission were excluded from the analysis. All databases were queried for entries indexed from 2000 onward.

### Screening process

**Figure 6:**
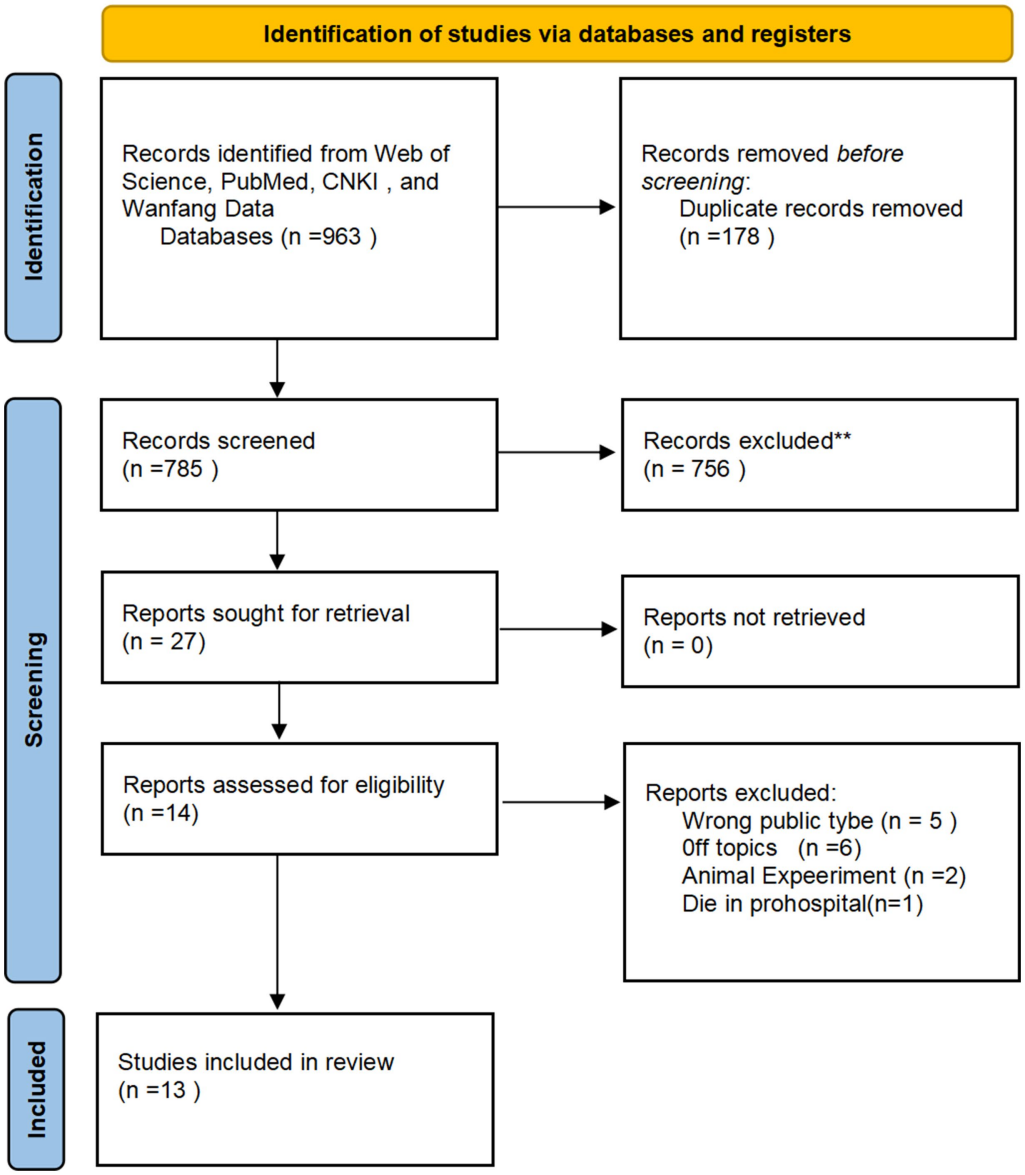
Work-flow of the screening and eligibility process of the systematic review according the PRISMA guidelines.

Following the search strategy across the designated databases, all retrieved records were exported into NoteExpress software for duplication remove. Two independent reviewers (Y.Z, B.S.) conducted an initial screening based on the titles and abstracts of all unique records. Full texts were obtained for all eligible articles. Excluded publications were categorized, specifying the reasons for exclusion (Figure 6).

The following data were recorded and collected: publication year, analytical methodology, patient cohort size, geographic provenance (province), age and sex distribution, therapeutic interventions administered, time to first medical contact, implicated food types, and outcome.

### Risk of bias assessment

Bias risk was assessed using the software package Review Manager (RevMan) software, Version 5.4 (The Cochrane Collaboration).and the OHAT guidelines. The final evaluation considered the following parameters: randomization sequence, allocation concealment, blinding of outcome assessment, completeness of procedure description, clarity of inclusion criteria, attrition bias, reporting bias, follow-up duration, and other biases. Bias risk was categorized as low, unclear, or high.

### Statistical analysis

Odds ratios (ORs) with corresponding 95% confidence intervals (CIs) were calculated for all reported outcomes using RevMan 5.4 (The Cochrane Collaboration). The Mantel–Haenszel method was applied under a random-effects model to account for anticipated cross-trial variations in study designs and intervention characteristics. Between-study heterogeneity was quantified using the I^2^ statistic in accordance with Cochrane Handbook guidelines.

## Result

### Publication screen

Database screening initially identified 963 publications. During the initial screening phase, 178 duplicate records were removed for duplication. Subsequently, 756 articles were excluded based on title and abstract evaluation. 27 articles proceeded to the eligibility assessment stage. Following full-text review, 14 publications were excluded from the final synthesis for the following reasons:5 articles were excluded due to off-topic,5 were review articles,2 involved animal or non-human subjects, One study was excluded because the subjects experienced prehospital mortality.

### Risk of bias assessment

**Figure 7:**
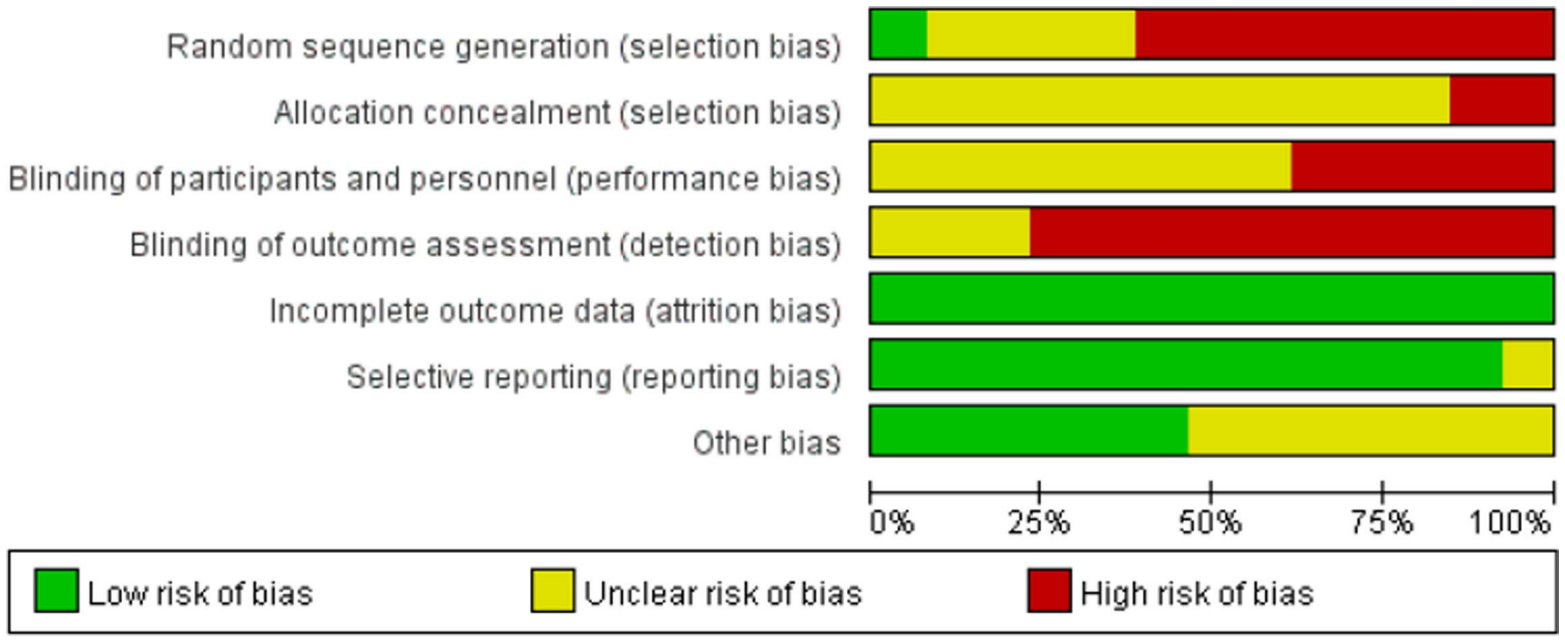
Risk of bias graph.

**Figure 8:**
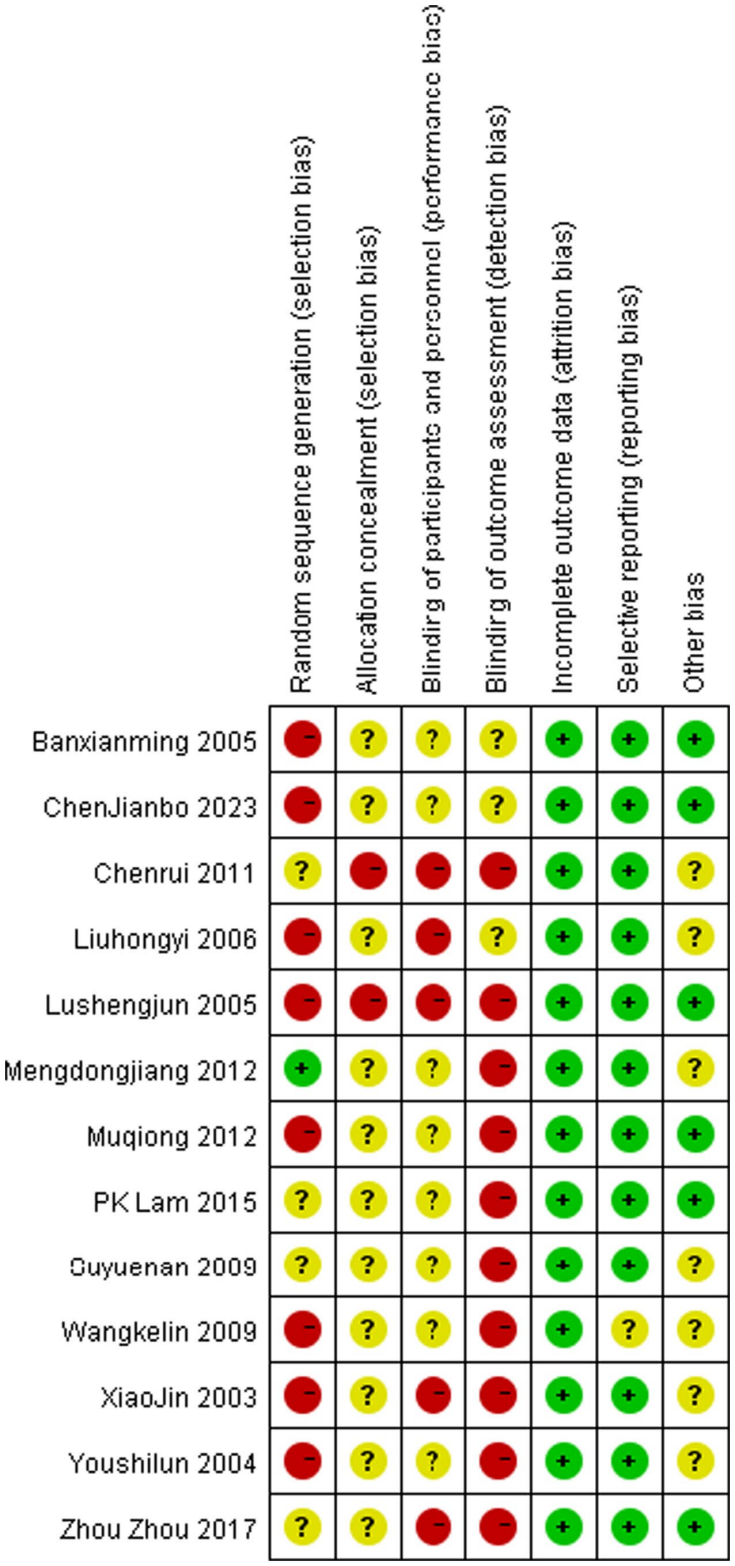
Risk of bias summary.

Risk of bias assessment for all included studies is presented in Figures 7, 8. All publications exhibited elevated bias risk (Figures 7, 8) attributable to extensive study design divergence, constrained observational periods, and abundant non-comparative clinical data.

### Study characteristics

A summary is depicted in the Table 3. Thirty fatalities occurred among 160 patients, yielding an in-hospital mortality rate of 18.75%. Among the thirteen case reports, six cases occurred in Guangdong, three in Guangxi, and one case each in Guizhou, Zhejiang, and Hong Kong SAR. The age distribution of cases is relatively wide, covering age groups including adolescents, middle-aged people, and the older adult. Due to the limited number of patients with clearly-defined gender data, a comprehensive gender-based analysis was not feasible. In all previous reports, no gender differences among deceased patients have been mentioned. According to summary of food type, the primary cause is accidental ingestion of soups, medicinal liquors containing liquids. Other causes include suicide and homicide (7, 8).

**Table 3:** Characteristics of studies included in systematic review (2000–2025).

Author, Year	*n*	Age	Province	Reason	Food type	Intervention	Time to FMC	Outcome
Ban, 2005 (9)	C = 4 E = 13	16–58 32.6 (average)	Guangxi	Suicide (13) Ingestion by mistake (4)	Unknow	Gastric lavage (13); Intravenous neostigmine infusion; Oxygen supplementation; Analeptic agents (1)	90 min (the death case)	Survival (16), Death (1)
Xiao et al., 2003 (10)	C = 0 E = 8	30–40	Guangdong	Ingestion by mistake	Soup	Gastric lavage; Symptom-targeted supportive; Mechanical ventilation (2)	Unknow	Survival (8)
You et al., 2004 (11)	C = 0 E = 13	25 (average)	Guangdong	Ingestion by mistake	Soup	Gastric lavage; Oxygen supplementation; Forced diuresis; Correction of acidosis	0.5-6 h	Survival (12), Death (1)
Wang et al., 2000 (12)	C = 0 E = 13	7–68	Guangdong	Ingestion by mistake (61) Suicide (8)	Soup (71), Unknow (8)	Gastrointestinal decontamination; Intravenous neostigmine infusion; Gastrointestinal decontamination	1-9 h	Survival (63), Death (15)
Su et al., 2009 (13)	C = 14 E = 6	32–75	Guangdong	Ingestion by mistake (19) Suicide (1)	Soup	Prophylactic endotracheal intubation; Gastric lavage (14); Catharsis induction; Hemoperfusion (1); Intravenous fluid;	Unknow	Survival (18), Death (2)
Liu, 2006 (30)	10	Unknow	Guangdong	Ingestion by mistake	Soup	Gastric lavage (6); Catharsis induction; Analeptic agents; Symptom-targeted supportive	1.25 h	Survival (6), Death (4)
Lu, 2005 (15)	C = 0 E = 1	18	Guangxi	Suicide	Soup	Endotracheal intubation; Gastric lavage; Intravenous atropine infusion; Intravenous neostigmine infusion	1 h	Survival
Chen et al. 2011 (7)	C = 0 E = 2	31,35	Guangxi	Suicide	Soup	Endotracheal intubation; Gastric lavage ; Intravenous neostigmine infusion	40-60 min	Survival (2)
Meng et al. 2012 (16)	C = 5 E = 3	Unknow	Guizhou	Ingestion by mistake	Medicinal liquor	Intravenous atropine infusion; Gastric lavage (3); Mechanical ventilation (3); Lidocaine bolus injection (1); CPR (2)	1.5 h	Survival (4), Death (4)
Mu, 2011 (17)	C = 5 E = 3	Unknow	Guizhou	Ingestion by mistake	Medicinal liquor	Mechanical ventilation; Epinephrine and vasopressor agents; Emesis induction; Gastric lavage; Hepatoprotective therapy	1–1.5 h	Survival (6), Long-term sequelae (1) Death (2)
Lam et al. 2015 (18)	C = 3 E = 0	19,53,54	Hong Kong	Ingestion by mistake	Soup	Intravenous fluid; Symptom-targeted supportive	11 h	Survival (3)
Zhou et al. 2017 (20)	C = 1 E = 0	26	Guangdong	Suicide	Soup	Mechanical ventilation; Symptom-targeted supportive	Unknow	Survival
Chen et al. 2023 (21)	C = 1 E = 1	75, Unknow	Zhejiang	Ingestion by mistake	Soup	CPR (1); Symptom-targeted supportive; Gastric lavage (1)	Unknow	Survival (1), PVS (1)
Total	C = 28 E = 132	--	Guangdong (6) Guangxi (3) Hong Kong (1) Guizhou (2) Zhejiang (1)	Suicide (25) Ingestion by mistake (166)	Soup (119); Medicinal liquor (16); Unknow (25)	--	--	Survival (130) Death or Long-term sequelae (30)

PVS, Persistent Vegetative State; CPR, Cardiopulmonary resuscitation; FMC,: First Medical Contact.

### Data statistic

**Figure 9:**
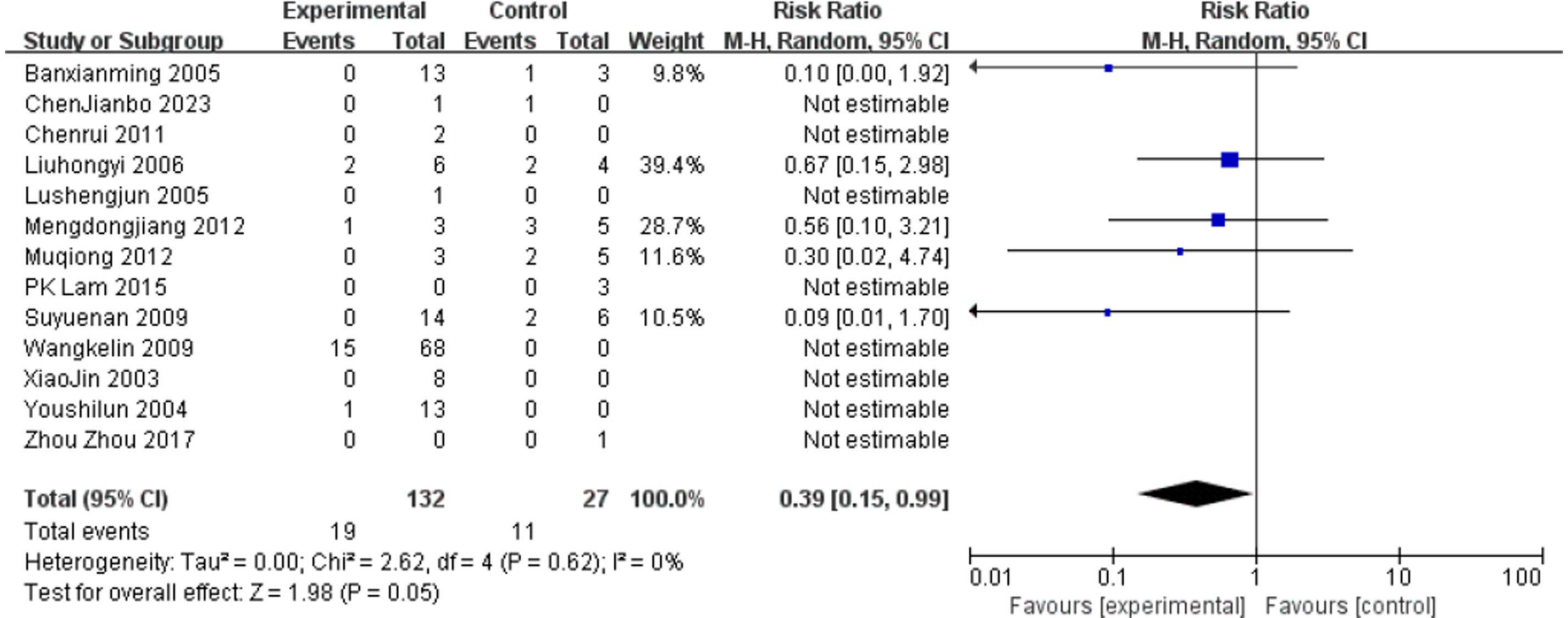
Forrset plot.

We used Mantel–Haenszel methods and forest plots to test if gastric lavage helps patients. Because age, toxin types, and other factors affect outcomes, we chose a random-effects model. The results surprised us: Lavage cut death rates by 61% versus controls (OR 0.39, 95% CI 0.15–0.99, *p* = 0.05). Studies agreed closely (I^2^ = 0%, Figure 9). We conducted a subgroup analysis of the clinical characteristics of 27 deceased patients for whom relatively detailed data were available (3, 7, 9–21).

## Discussion

According to phytochemical analyses, *G. elegans* plants contain 121 alkaloids and 25 iridoid compounds, which are primarily concentrated in the roots though also distributed in the entire plant (22). Pharmacological studies have shown that gelsemine, which is abundant in *G. elegans*, acts as a modulator of glycine receptors and type A GABA receptors in the central nervous system (CNS), causing CNS depression and respiratory and circulatory failure in poisoned individuals (23, 24). The main components of *G. elegans* alkaloids include gelsemicine, koumicine, kouminicine, and koumine with the most abundant alkaloid being koumine (25). Gelsenicine has the highest toxicity (LD 50 ~ 0.26 mg/kg rat, i.p.; and 0.15 mg/kg rat, i.v.), while gelsemine and koumine are also present in significant amounts (gelsemine, LD50 ~ 56 mg/kg mice, i.p.; koumine, LD50 ~ 100 mg/kg mice, i.p.) (22, 26).

This report describes three cases of acute poisoning resulting from the accidental ingestion of soup containing *G. elegans*. The patients presented with symptoms such as dizziness and shortness of breath, which rapidly deteriorated into respiratory failure and CNS depression within half an hour. The medical team administered treatments including endotracheal intubation, gastric lavage, hemoperfusion, and anti-microbial medications. They recovered due to timely respiratory support, and the pharmacologic treatment administered effectively reduced the risk of severe sequelae.

The conventional treatment approach includes respiratory support, gastric lavage, anti-emetics, blood perfusion, intravenous fluid therapy, and correction of acid–base imbalance and electrolyte disorders (20). Gastric lavage is a method for gastrointestinal decontamination following toxin ingestion, remains widely practiced in China (4). However, the clinical benefits derived by patients from this intervention are currently contentious. Another reason to avoid routine use involves toxins like gelsemine alkaloids absorb quickly through mucous membranes. Still, we have yet to clinically confirm this rapid-absorption theory. The animal studies showed that if gastric lavage was undertaken within 60 min, the mean recovery of marker was 13 and 8.6% (27). Among the included studies, two patients experienced cardiac arrest following gastric lavage (with one having recurrent episodes), though causality remains indeterminate between the procedure and underlying gelsemine alkaloid poisoning. Although data analysis showed an slight benefit, clinical significance remains uncertain due to uncontrolled variables like lavage fluid selection, severity variability, and treatment timing. Consequently, forest plot results failed to demonstrate significant clinical benefit. Recent studies suggest that endotracheal intubation should take precedence over gastric lavage due to the risk of respiratory failure from gelsemium poisoning and the need to ensure airway protection during gastric lavage (to reduce complications like aspiration pneumonia and cardiopulmonary arrest) (28). This approach may hold clinical value in treating gelsemium poisoning.

Excluding the 10 patients who died before reaching the hospital and the 8 cases without pre-hospital time-table information. Among those who died after consuming medicinal liquor, 81.25% were male. This phenomenon might be attributed to the traditional practice among middle-aged and older adult men in China of using medicinal liquor, they believed the herbal plant could tonify kidney from TCM theory.

There is currently no specific antidote for *G. elegans* poisoning. Some cases reports have noted that naloxone is particularly effective in rapidly alleviating respiratory depression and shortening the duration of patient unconsciousness, a mechanism believed to be related to its antagonism of enkephalin release induced by *G. elegans* poisoning (11). A 2022 study in mice found that the combination of flumazenil and epinephrine showed promising therapeutic effects in treating *G. elegans* poisoning (29). However, both therapies rely solely on limited preclinical data and isolated human cases, lacking robust evidence from large-scale clinical trials.

The main limitation of this study stems from the heterogeneous reporting format across the included clinical cases. Furthermore, nearly all poisoning reports lacked Poisoning Severity Score (PSS) data. This precluded meaningful subgroup analysis based on patient stratification and compromised the interpretation of the final results.

## Conclusion

*G. elegans* poisoning is a life-threatening condition that requires prompt clinical attention. It manifests as dizziness, respiratory depression, neuromuscular paralysis, and multi-organ failure, with a mortality rate of ~18.75%. Common symptoms of *G. elegans* poisoning include dizziness, chest tightness, respiratory depression, vertigo, nausea and vomiting (7, 8, 15). *G. elegans* alkaloids also inhibit spinal motor neurons, causing respiratory muscle paralysis. Gastric lavage may benefit patients presenting with acute poisoning, but its performance requires prior airway protection. Therefore, management should prioritize prompt respiratory support and airway protection. Although agents like naloxone and the combination of flumazenil with epinephrine show potential in alleviating symptoms (e.g., respiratory depression) based on limited preclinical and case report data, their efficacy lacks robust validation through large-scale clinical trials.
