# Correction: Caution on the severe damage of *Gelsemium elegans* poisoning: a case report on family poisoning and systematic review

**DOI:** 10.3389/fpubh.2025.1717881

**Published:** 2025-12-05

**Authors:** Yawen Zhao, Bisheng Shen, Qimei Xiao, Yameng Wang, Yi Fu, Qi Tang, Zhangrong Liang

**Affiliations:** 1 The Eighth Clinical Medical College of Guangzhou University of Chinese Medicine, Foshan, China; 2 Foshan Hospital of Traditional Chinese Medicine, Foshan, China

**Keywords:** *Gelsemium elegans*, poisoning, case report, review-systematic, gastric lavage

The **Article type** was erroneously chosen as Mini Review. The correct article type is Brief Research Report.

In the published article, there was a mistake in the **Keywords**. The keywords for this article were displayed as “*Gelsemium elegans*, poisoning, review-systematic, respiratory depression, gastric lavage”. Due to the change in the article type and its content, the keywords should be revised to: “*Gelsemium elegans*, poisoning, case report, review-systematic, gastric lavage”.

The original version of this article has been updated.

